# Assessment, detection, and validation of clinical associations of thirdhand smoke exposure (ADVOCATE) study protocol

**DOI:** 10.1038/s41390-025-03915-3

**Published:** 2025-02-13

**Authors:** E. Melinda Mahabee-Gittens, Georg E. Matt, Nicolas Lopez-Galvez, Eunha Hoh, Penelope J. E. Quintana, Nathan G. Dodder, Roman A. Jandarov, Lara Stone, Chase A. Wullenweber, Jasjit S. Ahluwalia, Ashley L. Merianos

**Affiliations:** 1 Divison of Emergency Medicine, Cincinnati Children’s Hospital Medical Center, Cincinnati, OH USA; 2 University of Cincinnati College of Medicine, Cincinnati, OH USA; 3 Department of Psychology, San Diego State University, San Diego, CA USA; 4 School of Public Health, San Diego State University, San Diego, CA USA; 5 San Diego State University Research Foundation, San Diego, CA USA; 6 Division of Biostatistics and Bioinformatics, Department of Environmental and Public Health Sciences, University of Cincinnati College of Medicine, Cincinnati, OH USA; 7 Department of Behavioral and Social Sciences, Brown University School of Public Health, Providence, RI USA; 8 School of Human Services, University of Cincinnati, Cincinnati, OH USA

## Abstract

**Background:**

Thirdhand smoke (THS) pollution is the residue of secondhand smoke (SHS) remaining in homes long after active smoking has ceased. This study is the first to characterize the clinical correlates of THS exposure (THSe) in children independent of secondhand smoke exposure (SHSe). The prevalence, sociodemographic characteristics, tobacco smoke exposure patterns, sources, clinical, and biomarker effects associated with THSe will be examined.

**Method:**

Smoking and nonsmoking parents and their 0–11-year-olds (*N* = 1013) were recruited. Children were categorized into tobacco smoke exposure (TSE) groups via biochemical validation with salivary cotinine and hand nicotine: (1) no exposure group (NEG); (2) THSe-only group (TEG); and (3) Mixed SHSe and THSe group (MEG). At enrollment, 6-weeks, and 6-months, parental assessments and children’s biological and home samples were obtained and analyzed for SHSe, THSe, THS pollution, inflammatory and oxidative stress markers.

**Results:**

The mean (SD) child age was 5.8 (3.4) years; 50.7% were female; and 97% were non-Hispanic (97.0%); 67.5% were White, 25.7% were Black, 6.8% were Other/unknown race. In total, 57.9%, 18.2%, and 21.9% were classified in the NEG, TEG, and MEG, respectively. Sample and data analyses are ongoing.

**Conclusion:**

This project will provide unique insights into how THSe in the absence of SHSe affects the clinical, inflammatory, and oxidative responses in children.

**Impact:**

- This is the first prospective longitudinal study to examine the prevalence of thirdhand smoke exposure in children of nonsmokers.
- Unlike prior tobacco smoke exposure research, this study will examine the contribution of thirdhand smoke exposure to pediatric health outcomes.
- Results will provide unique insights into how thirdhand smoke exposure in the absence of secondhand smoke exposure affects the clinical, inflammatory, and oxidative responses in children of nonsmokers.

## Introduction

Thirdhand smoke (THS) pollution is the chemical residue of secondhand smoke (SHS) found in dust, on surfaces, and embedded in furniture and building materials that persists long after active indoor smoking by former occupants has ceased.^1^ THS is often unnoticed and has been found in nonsmokers’ homes who have moved into homes previously occupied by smokers^2,3^ or who live in attached housing units in which tobacco smoking is actively occurring or has occurred in the past.^1,4,5^ THS also remains present in homes of smokers after they quit.^3^ THS pollution reservoirs are persistent and difficult to eliminate.^5,6^ Due to young children’s exploratory activities with floors, toys, furniture, and fabrics in their homes, they can be inadvertently exposed to THS pollutants via dermal absorption, ingestion, and inhalation.^1,4^ In addition, young children are 100 times more sensitive than adults to dust pollutants due to their smaller anatomical size, higher respiratory rate, and hand-to-mouth behaviors.^7,8^ The THS pollutants to which children may be exposed contain a mixture of chemicals known to have adverse pulmonary, cardiovascular, and neurological consequences,^9–12^ and are suspected of causing cancer and reproductive harm in humans.^1,4,13,14^

While there is no question that secondhand smoke exposure (SHSe) is causally linked to many pediatric illnesses,^12,15,16^ prior tobacco smoke exposure (TSE) research has ignored the contribution of exposure to THS residue to clinical outcomes. SHSe occurs via inhalation during and shortly after someone smokes near a child. Past research on TSE in general and SHS in children, in particular, did not consider exposure to THS residue and failed to compare the clinical findings observed in smokers’ children to groups of nonsmokers’ children who were not exposed to tobacco smoke, that is children who lived in homes free of SHS and THS.^17,18^ Rigorous human studies examining the prevalence and clinical effects of THS exposure (THSe) in children of nonsmokers have not been conducted. However, laboratory and nonhuman studies indicate that THSe may have concerning health effects and is associated with oxidative stress, genotoxicity, cytotoxicity, and carcinogenicity.^1,19–21^ Thus, there is a need for research examining the clinical effects of THSe in children of nonsmokers.^1^

Our team has found that even if smokers’ children live in homes with smoking bans, they may have high levels of THSe as evidenced by the presence of nicotine on their hands, a proxy measure for THS pollution in their environments.^17,18,22–24^ While this research also indicates that children of nonsmokers may have nicotine on their hands,^25^ the clinical consequences associated with THSe in nonsmokers’ children remain unknown. To fill this gap, we conducted a study called Assessment, Detection, and Validation of Clinical Associations of THSe (ADVOCATE), the first prospective observational longitudinal study to identify and examine children of nonsmokers who are exposed to THS. We recruited 0–11-year-old children and their parents and used strict parental screening and biochemical validation techniques using cotinine, the main nicotine metabolite,^26^ and hand wipe nicotine, a THS pollutant,^2,3,18,22^ to categorize and compare children in three TSE groups (*N* = 1013). The three groups were (1) No exposure group (NEG): lived with nonsmokers, no reported SHSe, confirmed negative levels of SHSe and THSe; (2) Thirdhand smoke exposure-only group (TEG): lived with nonsmokers, no reported SHSe, confirmed negative or low levels of SHSe, confirmed elevated levels of THSe; and (3) Mixed SHSe+THSe group (MEG): lived with smokers, reported SHSe, confirmed moderate or high levels of SHSe, confirmed high levels of THSe. In this study, the prevalence, sociodemographic characteristics, TSE patterns, sources, and clinical and biomarker effects associated with THSe will be examined. This manuscript describes the study protocol for ADVOCATE and the descriptive characteristics of the study sample overall.

## Methods/Design

### Study aims and hypotheses

The ADVOCATE study addresses three specific aims. Aim 1 is to conduct targeted screening and strict biochemical validation using cotinine and hand wipe nicotine levels to identify children in the three TSE groups (i.e., NEG, TEG, and MEG). The prevalence and biochemical levels of THSe and the sociodemographic characteristics, TSE patterns, and frequency of TSE-related illnesses (e.g., asthma), healthcare visits, and hospitalizations will be compared over time in each TSE group. Hypothesis 1.1 is that children with the highest levels of cotinine and hand nicotine will be in the MEG followed by the TEG, and they will more likely: be 2–4-years old; have been around more cigarettes; and live in multiunit, smaller, or older homes. Hypothesis 1.2 is that children in the MEG followed by those in the TEG will have the highest frequency of TSE-related illnesses, healthcare visits and hospitalizations compared to children in the NEG. Aim 2 is to conduct follow-up visits to assess the contribution of environmental TSE sources over 6-months to the THSe levels of children in each TSE group. Biological and home environmental samples will be assessed for TSE (urinary cotinine, 4-(methylnitrosamino)-1-(3-pyridyl)-1-butanol (NNAL)), THSe (urinary NNAL/cotinine ratio is a potential marker), THS pollution (hand wipe nicotine), and tobacco smoke pollution (air nicotine). Hypothesis 2.1 is that in TEG children, THSe will be higher than SHSe while in MEG children, SHSe will be higher than THSe. Hypothesis 2.2. is that the highest contribution to THSe levels will be from air, followed by dust. Aim 3 is to assess the association between THSe by group and biomarkers of inflammation (e.g., TNF-α) and oxidative stress (e.g., 8-isoprostane), pulmonary function, and clinical illness patterns over time. Hypothesis 3 is that children in the MEG followed by children in the TEG will have elevated biomarker responses to inflammation and oxidative stress and impaired pulmonary function compared to children in the NEG. Our exploratory hypothesis is that biomarker levels will be associated with TSE-related illnesses.

### Recruitment, setting, screening, and consent

Beginning in February 2020 through March 2023, research staff conducted targeted, standardized screening and recruitment of children and parents from the pediatric emergency department (PED) or Urgent Care (UC) at Cincinnati Children’s Hospital Medical Center (CCHMC). To enhance enrollment during the start of the COVID-19 pandemic, study flyers were created by CCHMC Marketing and sent via email or posted in locations at CCHMC to recruit participants. Additionally, a CCHMC Facebook page was created to advertise the study; both the flyers and the Facebook page provided a link that led potential participants through a preliminary screening process. Based on the screening responses, the participants were contacted by trained research staff to ask further screening questions. If eligible, the participants were then scheduled to come to the clinical research center to complete consent and study procedures. The CCHMC IRB approved this study, and parents signed informed consent for themselves and their children; children who were 11 years old signed assent. Consent included permission to collect and store additional and leftover samples in a secure repository for potential future research of this unique pediatric population of children with THSe, SHSe+THSe, and no TSE.

### Inclusion and exclusion criteria

Adult participants were required to meet the following inclusion criteria to be eligible for enrollment in the trial: (1) parents/legal guardian age ≥18 years; 2) if recruited from the PED/UC, the legal guardian had to be accompanying a child 0–11 years of age who presented to the PED/UC with a non-critical condition that did not require immediate care; (3) had lived in their home for at least six months; (4) were able to speak/read English; (5) had a permanent address and a working phone number; and (6) had no plans to move within the next nine months. For children of nonsmoking parents, additional eligibility included (7) having implemented a strict home and car smoking ban for the past >6 months (to exclude recent SHSe sources); (8) that the child had not been around anyone who smoked ≥4 times in the past month; and (9) parents or household members did not use cannabis.

Parent-child dyads were excluded if the: (1) children used tobacco, e-cigarettes, or cannabis; (2) children or parents were unable to complete assessments due to medical, cognitive, or other reasons; and (3) children were immunosuppressed or had malignancies. Notably, prior to recruitment, we planned to exclude parents who were exclusive electronic cigarette users due to differing emission types and use patterns, nicotine content, and exposure patterns. However, after the study started, we elected to include parents who were exclusive electronic cigarette users in the study so that we could conduct exploratory analyses of children who had exclusive secondhand aerosol exposure.

### Overall study procedures and progress to date

The overall objective is to identify the unique clinical correlates of THSe in children and the associations of THSe to specific illnesses and clinical and environmental markers. In Aim 1 of this prospective longitudinal cohort study, we conducted targeted screening with the goal of recruiting 1000 children whose parents were nonsmokers (goal was *N* = 800) and smokers (goal was *N* = 200). Once participants were enrolled, parents completed assessments, and study staff collected saliva and hand wipe samples from each child using standardized protocols. Samples were stored and batch shipped to the Environmental Health Laboratory at San Diego State University (SDSU) every three weeks and analyzed for salivary cotinine and hand wipe nicotine. Strict screening questions (Table 1) and strict biochemical criteria based on salivary cotinine (range ≤0.1 ng/ml for NEG to ≥1 ng/ml for MEG) and hand wipe nicotine levels (range <5 ng/wipe for NEG to >5 ng/wipe for MEG; see Table 2) were used to place children into either the NEG, TEG, or MEG. Parental assessments and electronic medical record (EMR) review were conducted at the baseline visit (T0) to examine sociodemographic characteristics, TSE patterns, and clinical information (e.g., diagnoses). In Aims 2 and 3, we conducted follow-up visits in a sequential sample of children validated to be in the NEG (*n* = 40), TEG (*n* = 40) and MEG (*n* = 40) at 6-weeks (T1), at 7-weeks (T2), at 6-months (T3), and at 6-months plus 1-week (T4). Parental assessments via email or phone (by preference) and EMR review (to verify parental responses) occurred on all participants at T1 and T3 to assess the frequency of TSE-related illnesses and healthcare visits. Thus far, all participants have been recruited, all parental assessments and samples collected, and study follow-ups are completed. Sample and data analyses and interpretation of results are ongoing.

**Table 1:** Targeted screening questions

	Nonsmokers goal: *N* = 800	Smokers goal: *N* = 200
Never smoked or nonsmoker for past year?	Yes	No
Strict home/car smoking ban ≥ 6 months?	Yes	No
Lives with smokers?	No	Yes
Parent(s) smokes at least 1 pack/week or 3 cigars/week?	No	Yes
Smoking occurs inside or outside the home (includes smoking bans)?	No	Yes
Occasional (<=3x/month) smoke exposure?	Yes or No	Yes or No

**Table 2:** Biochemical validation and categorization into TSE groups

Screening and biochemical validation	NEG goal: *N* = 560	TEG goal: *N* = 240	MEG goal: *N* = 200
T0 screen indicates parent is a nonsmoker?	Yes	Yes	No
Salivary cotinine level	≤ 0.1 ng/ml	>0.1 ng/ml but <1 ng/ml	≥1 ng/ml
Hand wipe nicotine level	< 5 ng/wipe	≥ 5 ng/wipe	>5 ng/wipe

*TSE* Tobacco smoke exposure, *NEG* no exposure group, *TEG* thirdhand smoke exposure-only group, *MEG* mixed secondhand smoke exposure + thirdhand smoke exposure group, *T0* baseline.

## Study procedures

### Targeted screening questions, sample collection, and TSE group categorization

After consent and enrollment, study staff used TSE screening questions^2,27,28^ (Table 1) to assess if children were in the nonsmoker group and would likely comprise the NEG or TEG once biochemical validation was completed (Table 2) or whether children were in the smoker group and would comprise the MEG. Based on prior research^2,18,28^ and the power analysis (See Power Analysis below), to ensure that we had adequate numbers of children in the TEG (at minimum 30% or *N* = 240), we estimated that we needed to recruit *N* = 800 nonsmokers and *N* = 200 smokers.^18,22^

Study staff collected saliva and hand wipe samples on all children which were shipped to and analyzed at SDSU. Rigorous biochemical validation using strict cutoffs of salivary cotinine and hand wipe nicotine were applied to place children into one of the TSE groups (Table 2).

## Assessment and retention procedures

### Parental assessments

At T0, parents completed electronic assessments, including sociodemographic characteristics and TSE patterns with questions on potential sources of SHSe and THSe. Exposure questions asked about smoked tobacco by parent and/or household members including type (e.g., cigarettes, cigars); e-cigarette and cannabis use; smoking restrictions in the home and family cars; number of smokers (e.g., household members, family, friends); number of cigarettes/e-cigarettes smoked/vaped around the child in the past week in the home, car, or other environments; housing rules about smoking in indoor or outdoor common areas, balconies, or porches (if child lived in multiunit housing). To assess potential THS sources in microenvironments that the child is in, we assessed the home environment, including home age, length of time lived in the home, history of former smokers in the home, home size, number of rooms and windows, floor types and surfaces, cleaning and vacuuming habits including the use of cleaning agents, ventilation, air conditioning, and air purifier use, and type of heat and stoves.

### EMR review and follow-up

An EMR review was conducted at T0 to assess chief complaints and disposition, and whether the child had a TSE-related medical history or discharge diagnosis. Specific ICD-10 diagnoses of TSE-illnesses were used based on prior work and the U.S. Surgeon General Reports.^15,29,30^ Email or phone parental assessments were conducted based on parents’ preference at T1 and T3 to assess if there had been any TSE-related: illnesses; sick-days; missed school/work days; and primary care or UC visits. EMR reviews at T2 and T4 were conducted to determine if there were any CCHMC-specific PED/UC visits and hospitalizations. See Table 3.

**Table 3:** Measures, sample collection and analyses by assessment point

	T0	T1	T2	T3	T4
Parental Assessments: Demographics, TSE patterns; TSE-related illnesses and visits	**✓**	**✓**		**✓**	
EMR Review: TSE-related: past medical history, diagnoses, PED/UC visits, and hospitalizations	**✓**		**✓**		**✓**
Saliva: Marker of overall TSE - Cotinine	**✓**	**✓**		**✓**	
Hand wipes: Marker of THS pollution in the environment - Nicotine	**✓**	**✓**		**✓**	
Silicone Wristbands: Marker of Tobacco Smoke Pollution in the environment - Nicotine			**✓**		**✓**
Urine: Markers of TSE and THS – Cotinine and NNAL	**✓**	**✓**		**✓**	
Saliva: Markers of immune response - Cytokines IL-8, IL-10, IL-1β, and TNF-α	**✓**	**✓**		**✓**	
Urine: Oxidative stress biomarkers: 8-isoprostane	**✓**	**✓**		**✓**	
Pulmonary Function Tests: FVC, FEV1 (age >5)		**✓**		**✓**	
Dust: THS Pollution- Nicotine			**✓**		**✓**

*T0* baseline, *T1* 6-weeks, *T2* 7-weeks, *T3* 6-months, *T4* 6-months+1 week, *TSE* tobacco smoke exposure, *EMR* electronic medical record, *PED* pediatric emergency department, *UC* Urgent Care, *THS* thirdhand smoke, *NNAL* 4-(methylnitrosamino)-1-(3-pyridyl)-1-butanol, *FVC* forced vital capacity, *FEV1* forced expiratory volume in one second.

#### Participant retention strategies

To facilitate recruitment and retention, many strategies were employed, including increasing incentives for each visit, the option to conduct home visits at T1-T4, multiple contact efforts to schedule follow-up visits, sending reminder letters and emails prior to visits, and options to complete assessments via email.

## Sample collection and chemical analysis

### Overview of sample collection, handling, and storage

#### Sample collection

Standardized protocols were used to collect all data and samples at the T0 visit and at T1-T4 follow-up visits. We collected the following child samples: saliva, urine, hand wipes, and silicone wristbands. Saliva samples were obtained by having the child spit on or suck cotton swabs for >1 min until moist; swabs were placed in a Salimetrics test tube and centrifuged for 15 min. Urine samples were collected by having the child urinate into a cup (if potty trained) or by using a syringe to extract urine from surgical cotton cloths in the diapers of children (if not potty trained). Hand wipes were obtained using a procedure established by us and used with children and adults.^24^ Briefly, hand wipes were collected by wiping the palms and volar surfaces of the fingers of children’s dominant hand with prescreened cotton swabs that were wetted with a solution of distilled water and 0.1% ascorbic acid; field blanks were collected to adjust for potential environmental contaminants.^31^ The surface area of children’s hands was measured with calibers and parents were asked when their children’s hands were last washed. Hand wipes were placed in baked amber jars. Silicone wristbands were obtained from 24hourwristbands.com and cleaned in the laboratory prior to use. Wristbands were placed on children during the T1 and T3 visits, worn for one week, and then retrieved at follow-up visits at T2 and T4. Wristband field blanks were also collected for all participants. Participants were instructed to wear the wristbands at all times, including when sleeping, bathing, or swimming. In addition, patients age ≥5 years had pulmonary function tests measured at T1 and T3 with the NDD EasyOne portable spirometer. After instruction by the study staff, the best of at least three attempts were recorded; results were expressed as a percentage of predicted reference values.^32^ The PFTs measured were forced vital capacity (FVC) and forced expiratory volume in one second (FEV1). Within-person changes in PFTs were calculated by subtracting values between time points; within-person changes and between-group differences were expressed as absolute differences in percent.^33^

With regards to house dust samples, parents were asked to give study staff their vacuum bags at T2 and T4. Dust from the vacuum bags were transferred to a methanol-washed amber vial by study staff to collect approximately 1 cm of dust.

All samples were placed on ice and stored at −80 °C until ready for shipment; saliva samples were centrifuged and then frozen. Samples were shipped on dry ice overnight via Federal Express to: SDSU (saliva for cotinine, and hand wipes, dust, and wristbands for nicotine, Dr. Hoh’s laboratory); University of California at Riverside (urine for oxidative stress markers, Dr. Talbot’s laboratory); the Clinical Pharmacology Laboratory at University of California at San Francisco (urine for nicotine metabolites, Dr. Jacob’s lab); and the University of Minnesota (saliva for inflammatory markers, Dr. Gross’s lab). Once at these laboratories, samples were stored at −20 °C until ready for analysis.

#### San Diego State University analyses: salivary cotinine, hand wipe nicotine, dust nicotine, and wristband nicotine

Samples were prepared for instrumental analysis using extraction methods based on QuEChERS.^34,35^ The hand wipe nicotine,^24^ dust nicotine,^6^ and wristband nicotine^36^ methods were described previously, with the following addition to the wristband method. Prior to deployment, all wristbands were solvent-rinsed in the following sequence: twice with water, followed by 1 Molar KOH, methanol, hexane, and acetone. At each step, the beaker was gently rotated on an orbital shaker for 30 min.

Saliva was prepared for cotinine analysis using a similar QuEChERS procedure, as follows. All containers and syringes were rinsed with methanol or acetonitrile before use, and disposable caps and laboratory coats were worn by personnel during the sample preparation. Samples were thawed, and 0.5 mL of saliva was combined with 0.2 mL acetone, spiked with 2 ng of cotinine-*d*_*3*_, and equilibrated for 15 min. Two mL acetonitrile was added, followed by 0.8 g magnesium sulfate and 0.2 g sodium chloride. The samples were vortexed for 1 min between each of the previous steps. Samples were centrifuged at 3000 rpm (900 × *g*) and the supernatant (organic) layer was passed through a PTFE syringe filter (13 mm diameter, 0.2 µm pore size). The final concentration of cotinine-*d*_*3*_ was 5 ng/mL. The samples were evaporated to 0.1 mL and reconstituted to 0.4 mL of 5% acetonitrile and 95% water, the initial mobile phase of the instrumental method. If less than 0.5 mL of saliva was available, the volume was measured and used for conversion to units of ng cotinine/mL saliva.

For all sample types, nicotine or cotinine was quantified by separate isotope-dilution liquid chromatography tandem mass spectrometry (LC-MS/MS) methods as described in Quintana et al.^36^ Briefly, the internal standards were nicotine-*d*_*4*_ or cotinine-*d*_*3*_, the LC separation for nicotine used hydrophilic interaction liquid chromatography (HILIC), and the separation for cotinine used C_18_ reverse phase chromatography. Both methods used positive electrospray ionization and multiple-reaction-monitoring (MRM). The calibration curves were generated using external standard solutions. Values for the limit of quantification (LOQ)^37^ and the estimated method detection limit (MDL)^38^ were determined for each analytical method. Quality assurance/quality control (QA/QC) procedures included field and laboratory blank analysis, monitoring for LC injection carryover, and ensuring the stability of the internal standard recovery and instrumental sensitivity across samples. All MRM chromatographic peak areas and quantification results were exported to custom semi-automated software to verify the QA/QC criteria and ensure the data reporting was consistent from batch-to-batch throughout the study timeframe.

The hand wipe nicotine MDL was 0.19 ng/sample and the LOQ was 0.30 ng nicotine/sample, with the values normalized to ng nicotine/cm^2^. The dust nicotine MDL was 0.13 ng/sample and the MDL was 0.20 ng/sample, with the values normalized to ng nicotine/g dust (concentration) and ng nicotine/m^3^ (loading). The wristband nicotine MDL was 0.19 ng/sample and the LOQ was 0.30 ng/sample, with the values normalized to ng nicotine/g wristband. The salivary cotinine MDL was 0.008 ng/sample and the LOQ was 0.04 ng cotinine/sample, with the values normalized to ng cotinine/mL saliva. The reporting limits were equal to the MDLs.

#### University of Minnesota saliva analyses: inflammatory markers

IL-8, IL-10, IL-1β, and TNF-α were assayed in saliva using a commercially available Simple Plex immunoassay 4-plex on the ELLA automated immunoassay system (Bio-Techne, San Jose, CA) according to the manufacturer’s instructions.^39^ Simple Plex has shown excellent correction with conventional immunoassays including ELISA.^40^

#### University of California at Riverside urine analyses: oxidative stress marker 8-isoprostane

The Detroit R&D 8-isoprostane Oxidative Stress ELISA Kit (Detroit, Michigan)^41,42^ was used to determine 8-isoprostane concentration in urine samples. A 1:4 dilution using the diluent provided with the kit was used. The color intensity of each sample was measured using Epoch micro-plate reader (Agilent Technologies, Santa Clara, CA) at a wavelength of 450 nm.

#### University of California at San Francisco urine analyses: TSE metabolites

Urinary cotinine concentrations were determined using LC-MS/MS by the method of Jacob et al.^43^ with an LOQ of 0.05 ng/mL. Urinary NNAL concentrations were determined using LC-MS/MS by the method of Jacob et al.^44^ with an LOQ of 0.25 pg/mL. The reporting limits were equal to the LOQs.

## Statistical analysis

### Power analysis and sample size calculations

We conducted separate power analyses for the three scientific aims based on effect size estimates from previous studies. We expected that the NEG, TEG, and MEG groups would account for 3–7% of the overall variance (R^2^ = 0.25–0.40) in TSE and THSe.^17,18,22–25^ Therefore, sample sizes were based on achieving power of 0.80 to detect a partial R^2^ = 0.03–0.07 in a model with an overall R^2^ = 0.25–0.40. In summary, it was determined that a total *n* of 244 (~81 per group) was required to achieve 80% power on a conservative scenario (partial r^2^ = 0.03 and R^2^ = 0.25). For the most optimistic scenario (partial r^2^ = 0.07 and R^2^ = 0.4), a total *n o*f 86 (~28 or 29 per group) was adequate. Type I error was set at 0.05. Thus, a total sample size of *N* = 120 (i.e., *N* = 40 in each of NEG, TEG, MEG) yielded power >0.80 across plausible effect size scenarios for all three aims.

### Statistical analysis

Statistical analyses are based on the general linear model using log-transformed continuous and nominal outcome variables. Based on the distributional and measurement characteristics, we will employ various regression models, including ordinary least squares, maximum likelihood for uncensored data, left-censored models, hurdle models, as well as ordinal, logistic, multinomial, and accelerated failure time models. A multiple linear regression model will be developed for each THSe outcome, as well as a multivariate multiple linear regression model to jointly model cotinine and hand nicotine and their associations with TSE groups. All multiple regression models will control for potential covariates of clinical interest or covariates that are statistically significantly associated with the outcome, such as child age, number of cigarettes smoked around the child, and type, size, and age of home, as well as potential interaction terms. There is some evidence in the literature concerning racial/ethnic differences in nicotine metabolism.^45,46^ Thus, we will include racial/ethnic background in our models when analyzing nicotine and related outcomes. Model diagnostics will be performed to determine whether all model assumptions are valid. Maximum likelihood estimation methods for left-censored data based on Tobit or AFT model will also be explored.

We will follow guidelines in Little et al.,^47^ to minimize attrition and optimize the use of all data. Additionally, we will examine potential biases introduced through selection and attrition by examining differences between participants who did and did not enroll and between participants who were lost to follow-up and those retained. We will examine how attrition may affect generalizability; statistical models may require adjustment to control for biases.

### Characteristics of the study population

In total, 1004 children were categorized into TSE groups, and the mean (SD) age of children overall was 5.8 (3.4) years. About half (50.7%) of children were female, and the majority were non-Hispanic (97.0%) and White (67.5%), followed by 25.7% Black, 5.0% Other race including Multiracial, and 1.8% were unknown race. A total of 57.9% of children were classified in the NEG, 18.2% were classified in the TEG, 21.9% were classified in the MEG, and 1.8% were classified in the secondhand aerosol exposure group. The Geometric Mean (GeoM; 95% CI for hand nicotine (ng nicotine/wipe) for the NEG, TEG, and MEG was: (1.19; [1.11, 1.28]), (11.60; [10.23; 13.14]), and (61.50; [50.41, 74.99]), respectively. The GeoM; 95% CI for salivary cotinine (*N* = 930; ng/mL) for the NEG, TEG, and MEG was (0.06; [0.05, 0.07]), (0.37; [0.28, 0.47]), and (3.73; [3.12, 4.43]), respectively. Please see Table 4 for additional details of the study sample overall and by TSE group status.

**Table 4:** Sociodemographic characteristics of children and parents in the ADVOCATE study

	Overall
	(*N* = 1004)
Characteristic	*n* (%)^a^
Child age, *M* (SD)	5.8 (3.4)
Child sex	
Male	495 (49.3)
Female	509 (50.7)
Child ethnicity	
Non-Hispanic	971 (97.0)
Hispanic	30 (3.0)
Child race	
Non-Hispanic White	678 (67.5)
Non-Hispanic Black	258 (25.7)
Non-Hispanic other/multiracial/uknown	50 (6.8)
Caregiver age, *M* (SD)	35.2 (7.6)
Caregiver sex	
Male	157 (15.6)
Female	847 (84.4)
Caregiver ethnicity	
Non-Hispanic	957 (95.5)
Hispanic	45 (4.5)
Caregiver race (*n* = 1003)	
Non-Hispanic White	715 (71.3)
Non-Hispanic Black	218 (21.7)
Non-Hispanic other/multiracial	70 (7.0)
Caregiver education level	
≤High school graduate/equivalent	227 (22.6)
Some college	180 (17.9)
College graduate	296 (29.5)
Post-graduate	301 (30.0)
Household Income	
<$30,000	268 (26.7)
$30,001–$90,000	325 (32.5)
>$90,000	409 (40.8)

*TSE* tobacco smoke exposure, *NEG* no TSE group, *TEG* thirdhand smoke exposure group, *MEG* mixed secondhand and thirdhand smoke exposure group.

^a^Percent refers to valid percent unless noted otherwise.

## Discussion

There is a wealth of research that causally links SHSe to many pediatric illnesses.^12,15,16^ While it is suspected that exposure to THS residue is associated with many of the same illnesses as SHSe, in the absence of large-scale, well-controlled human studies which include children of smokers who are exposed to both SHS and THS and children of nonsmokers who have no TSE or who are exposed to THS only, conclusions about the clinical effects of THSe on children cannot be made.^1,4^ Thus, this project is crucial to extend laboratory findings to pediatric clinical settings, examining clinical outcomes in children living in real-world environments. Specifically, this will be the first project to use strict screening measures and biochemical validation to identify and verify the TSE status of children so that a unique cohort of children with THSe only can be examined and compared to children with mixed SHSe+THSe and an unexposed group of children with no TSE. Among this group of unexposed children, we have identified 183 (18.2%) children with THS exposure who comprise the unique TEG. We have longitudinally followed this cohort using repeated assessments and biomarker measures to: (1) assess if there are differences in TSE-related illnesses in children in the TEG compared to those in the MEG and NEG; (2) discriminate the inflammatory and oxidative stress biomarker responses and pulmonary function test results in children exposed to THS in the TEG compared to children in the MEG and NEG; (3) examine the contribution of multiple environmental sources of THS pollution over time on the levels of THSe in children; and (4) use a novel sampling device, a silicone wristband, to measure airborne tobacco smoke pollution.

This project will enhance prior research findings by providing validated clinical correlates of exposure to THS residue in a pediatric population investigated in real-world field settings. Results will shed light on clinical effects attributable to THS, novel interventions to reduce childhood TSE, policy efforts needed to address persistent tobacco-related health disparities in housing, and to ensure better protection of children from TSE. The findings have the potential to result in a new understanding of the pre-clinical and clinical effects of THSe on children and to improve and expand existing preventive measures.

## Data Availability

Upon completion, the de-identified datasets obtained from the current study will be available from the corresponding author on reasonable request.

## References

[1] Matt, G. E. et al. Policy-Relevant Differences between Secondhand and Thirdhand Smoke: Strengthening Protections from Involuntary Exposure to Tobacco Smoke Pollutants. *Tob. Control* **33**, 798–806 (2024). 37263783 10.1136/tc-2023-057971 PMC11503167

[2] Matt, G. E. et al. When Smokers Move out and Non-Smokers Move In: Residential Thirdhand Smoke Pollution and Exposure. *Tob. Control* **20**, e1 (2011). 21037269 10.1136/tc.2010.037382 PMC3666918

[3] Matt, G. E. et al. When Smokers Quit: Exposure to Nicotine and Carcinogens Persists from Thirdhand Smoke Pollution. *Tob. Control* **26**, 548–556 (2016). 27655249 10.1136/tobaccocontrol-2016-053119 PMC6478602

[4] Jacob, P. et al. Thirdhand Smoke: New Evidence, Challenges, and Future Directions. *Chem. Res. Toxicol.* **30**, 270–294 (2017). 28001376 10.1021/acs.chemrestox.6b00343 PMC5501723

[5] Matt, G. E. et al. Persistent Tobacco Smoke Residue in Multiunit Housing: Legacy of Permissive Indoor Smoking Policies and Challenges in the Implementation of Smoking Bans. *Prev. Med. Rep.* **18**, 101088 (2020). 32368436 10.1016/j.pmedr.2020.101088 PMC7186560

[6] Matt, G. E. et al. Remediating Thirdhand Smoke Pollution in Multiunit Housing: Temporary Reductions and the Challenges of Persistent Reservoirs. *Nicotine Tob. Res.* **23**, 364–372 (2021). 32803265 10.1093/ntr/ntaa151 PMC7822102

[7] Northrup, T. F. et al. Thirdhand Smoke: State of the Science and a Call for Policy Expansion. *Public Health Rep* **131**, 233–238 (2016). 26957657 10.1177/003335491613100206 PMC4765971

[8] Roberts, J. W. et al. Monitoring and Reducing Exposure of Infants to Pollutants in House Dust. *Rev. Environ. Contam. Toxicol.* **201**, 1–39 (2009). 19484587 10.1007/978-1-4419-0032-6_1

[9] Castro, E. M., Lotfipour, S. & Leslie, F. M. Nicotine on the Developing Brain. *Pharmacol. Res.* **190**, 106716 (2023). 36868366 10.1016/j.phrs.2023.106716 PMC10392865

[10] Ahmad, S. et al. Acute Pulmonary Effects of Aerosolized Nicotine. *Am. J. Physiol. Lung Cell Mol. Physiol.* **316**, L94–L104 (2019). 30358437 10.1152/ajplung.00564.2017 PMC6383503

[11] McGrath-Morrow, S. A. et al. The Effects of Nicotine on Development. *Pediatrics* **145**, e20191346 (2020). 10.1542/peds.2019-1346 PMC7049940 32047098

[12] Raghuveer, G. et al. Cardiovascular Consequences of Childhood Secondhand Tobacco Smoke Exposure: Prevailing Evidence, Burden, and Racial and Socioeconomic Disparities: A Scientific Statement from the American Heart Association. *Circulation* **134**, e336–e359 (2016). 27619923 10.1161/CIR.0000000000000443 PMC5207215

[13] Matt, G. E. et al. Thirdhand Tobacco Smoke: Emerging Evidence and Arguments for a Multidisciplinary Research Agenda. *Environ. Health Perspect.* **119**, 1218–1226 (2011). 21628107 10.1289/ehp.1103500 PMC3230406

[14] Tang, X. et al. Thirdhand Exposures to Tobacco-Specific Nitrosamines through Inhalation, Dust Ingestion, Dermal Uptake, and Epidermal Chemistry. *Environ. Sci. Technol.* **56**, 12506–12516 (2022). 35900278 10.1021/acs.est.2c02559 PMC11439435

[15] U.S. Department of Health and Human Services. *The Health Consequences of Smoking: 50 Years of Progress. A Report of the Surgeon General* (U.S. Department of Health and Human Services, Centers for Disease Control and Prevention, National Center for Chronic Disease Prevention and Health Promotion, Office on Smoking and Health, 2014).

[16] Best, D. Committee on Environmental Health, Committee on Native American Child Health & Committee on Adolescence. From the American Academy of Pediatrics: Technical Report-Secondhand and Prenatal Tobacco Smoke Exposure. *Pediatrics* **124**, e1017–e1044 (2009). 10.1542/peds.2009-2120 19841110

[17] Mahabee-Gittens, E. M. et al. Differential Associations of Hand Nicotine and Urinary Cotinine with Children’s Exposure to Tobacco Smoke and Clinical Outcomes. *Environ. Res.* **202**, 111722 (2021). 34297932 10.1016/j.envres.2021.111722 PMC8578289

[18] Mahabee-Gittens, E. M., Merianos, A. L., Hoh, E., Quintana, P. J. & Matt, G. E. Nicotine on Children’s Hands: Limited Protection of Smoking Bans and Initial Clinical Findings. *Tob. Use Insights* **12**, 1179173X18823493 (2019). 30728727 10.1177/1179173X18823493 PMC6351963

[19] Hang, B. et al. Thirdhand Smoke: Genotoxicity and Carcinogenic Potential. *Chronic Dis. Transl. Med.* **6**, 27–34 (2020). 32226932 10.1016/j.cdtm.2019.08.002 PMC7096322

[20] Sarker, A. H. & Hang, B. Tobacco-Specific Nitrosamine 1-(N-Methyl-N-Nitrosamino)-1-(3-Pyridinyl)-4-Butanal (Nna) Causes DNA Damage and Impaired Replication/Transcription in Human Lung Cells. *PLoS One* **17**, e0267839 (2022). 35576221 10.1371/journal.pone.0267839 PMC9109921

[21] Diez-Izquierdo, A. et al. Update on Thirdhand Smoke: A Comprehensive Systematic Review. *Environ. Res.* **167**, 341–371 (2018). 30096604 10.1016/j.envres.2018.07.020

[22] Mahabee-Gittens, E. M., Merianos, A. L. & Matt, G. E. Preliminary Evidence That High Levels of Nicotine on Children’s Hands May Contribute to Overall Tobacco Smoke Exposure. *Tob. Control* **27**, 217–219 (2018). 28360145 10.1136/tobaccocontrol-2016-053602 PMC5623162

[23] Mahabee-Gittens, E. M. et al. Hand Nicotine as an Independent Marker of Thirdhand Smoke Pollution in Children’s Environments. *Sci. Total Environ.* **849**, 157914 (2022). 35952873 10.1016/j.scitotenv.2022.157914 PMC10199779

[24] Matt, G. E. et al. Changes and Stability of Hand Nicotine Levels in Children of Smokers: Associations with Urinary Biomarkers, Reported Child Tobacco Smoke Exposure, and Home Smoking Bans. *Environ. Int.* **181**, 108239 (2023). 37852151 10.1016/j.envint.2023.108239 PMC11678324

[25] Matt, G. E. et al. Prevalence and Income-Related Disparities in Thirdhand Smoke Exposure to Children. *JAMA Netw. Open* **5**, e2147184 (2022). 35129597 10.1001/jamanetworkopen.2021.47184 PMC8822372

[26] Benowitz, N. L. et al. Biochemical Verification of Tobacco Use and Abstinence: 2019 Update. *Nicotine Tob. Res.* **22**, 1086–1097 (2020). 31570931 10.1093/ntr/ntz132 PMC7882145

[27] Matt, G. E. et al. Households Contaminated by Environmental Tobacco Smoke: Sources of Infant Exposures. *Tob. Control* **13**, 29–37 (2004). 14985592 10.1136/tc.2003.003889 PMC1747815

[28] Mahabee-Gittens, E. M. et al. Contribution of thirdhand smoke to overall tobacco smoke exposure in pediatric patients: study protocol. *BMC Public Health* **19**, 491 (2019). 10.1186/s12889-019-6829-7 PMC6498613 31046729

[29] Office on Smoking and Health (US). *The Health Consequences of Involuntary Exposure to Tobacco Smoke: A Report of the Surgeon General* (Centers for Disease Control and Prevention (US), 2006). 20669524

[30] Mahabee-Gittens, E. M. et al. Healthy Families: Study Protocol for a Randomized Controlled Trial of a Screening, Brief Intervention, and Referral to Treatment Intervention for Caregivers to Reduce Secondhand Smoke Exposure among Pediatric Emergency Patients. *BMC Public Health* **17**, 374 (2017). 28464887 10.1186/s12889-017-4278-8 PMC5414142

[31] Quintana, P. J. et al. Wipe Sampling for Nicotine as a Marker of Thirdhand Tobacco Smoke Contamination on Surfaces in Homes, Cars, and Hotels. *Nicotine Tob. Res.* **15**, 1555–1563 (2013). 23460657 10.1093/ntr/ntt014

[32] Knudson, R. J., Lebowitz, M. D., Holberg, C. J. & Burrows, B. Changes in the Normal Maximal Expiratory Flow-Volume Curve with Growth and Aging. *Am. Rev. Respir. Dis.* **127**, 725–734 (1983). 6859656 10.1164/arrd.1983.127.6.725

[33] O’Rourke, J. M., Kalish, L. A., McDaniel, S. & Lyons, B. The Effects of Exposure to Environmental Tobacco Smoke on Pulmonary Function in Children Undergoing Anesthesia for Minor Surgery. *Paediatr. Anaesth.* **16**, 560–567 (2006). 16677267 10.1111/j.1460-9592.2005.01821.x

[34] Paya, P. et al. Analysis of Pesticide Residues Using the Quick Easy Cheap Effective Rugged and Safe (Quechers) Pesticide Multiresidue Method in Combination with Gas and Liquid Chromatography and Tandem Mass Spectrometric Detection. *Anal. BioAnal. Chem.* **389**, 1697–1714 (2007). 17909760 10.1007/s00216-007-1610-7

[35] Lehotay, S. J., de Kok, A., Hiemstra, M. & Van Bodegraven, P. Validation of a Fast and Easy Method for the Determination of Residues from 229 Pesticides in Fruits and Vegetables Using Gas and Liquid Chromatography and Mass Spectrometric Detection. *J. AOAC Int.* **88**, 595–614 (2005). 15859089

[36] Quintana, P. J. E. et al. Nicotine Levels in Silicone Wristband Samplers Worn by Children Exposed to Secondhand Smoke and Electronic Cigarette Vapor Are Highly Correlated with Child’s Urinary Cotinine. *J. Expo. Sci. Environ. Epidemiol.* **29**, 733–741 (2019). 30728487 10.1038/s41370-019-0116-7

[37] Armbruster, D. A. & Pry, T. Limit of Blank, Limit of Detection and Limit of Quantitation. *Clin. Biochem. Rev.* **29**, S49–S52 (2008). 18852857 PMC2556583

[38] U.S. Federal Government. Code of Federal Regulations. 40 Cfr Appendix B to Part 136 - Definition and Procedure for the Determination of the Method Detection Limit-Revision 1.11. In *Title 40 - Protection of Environment* (Authenticated U.S. Government Information, 2011).

[39] Bio-Techne. *Simple Plex™ Assays*, https://www.bio-techne.com/reagents/simple-plex-immunoassays/assay-menu. Accessed January 17, 2024.

[40] Aldo, P., Marusov, G., Svancara, D., David, J. & Mor, G. Simple Plex() : A Novel Multi-Analyte, Automated Microfluidic Immunoassay Platform for the Detection of Human and Mouse Cytokines and Chemokines. *Am. J. Reprod. Immunol.* **75**, 678–693 (2016). 27170460 10.1111/aji.12512 PMC5084752

[41] Detroit R. & D Inc. *8-Isoprostane Elisa Kit*, https://www.detroitrandd.com/sites/default/files/documents/8_iso_spec_sheet_10-31-23.pdf. Accessed January 17, 2024.

[42] Sakamaki-Ching, S. et al. Correlation between Biomarkers of Exposure, Effect and Potential Harm in the Urine of Electronic Cigarette Users. *BMJ Open Respir. Res*. **7**, e000452 (2020). 10.1136/bmjresp-2019-000452 PMC7047495 32079607

[43] Jacob, P. et al. Determination of the Nicotine Metabolites Cotinine and Trans-3’-Hydroxycotinine in Biologic Fluids of Smokers and Non-Smokers Using Liquid Chromatography-Tandem Mass Spectrometry: Biomarkers for Tobacco Smoke Exposure and for Phenotyping Cytochrome P450 2a6 Activity. *J. Chromatogr. B. Analyt. Technol. Biomed. Life Sci* **879**, 267–276 (2011). 21208832 10.1016/j.jchromb.2010.12.012 PMC3050598

[44] Jacob, P. et al. Subpicogram Per Milliliter Determination of the Tobacco-Specific Carcinogen Metabolite 4-(Methylnitrosamino)-1-(3-Pyridyl)-1-Butanol in Human Urine Using Liquid Chromatography-Tandem Mass Spectrometry. *Anal. Chem.* **80**, 8115–8121 (2008). 18841944 10.1021/ac8009005 PMC3167662

[45] Murphy, S. E. Nicotine Metabolism and Smoking: Ethnic Differences in the Role of P450 2a6. *Chem. Res. Toxicol.* **30**, 410–419 (2017). 28092945 10.1021/acs.chemrestox.6b00387 PMC8205229

[46] Wilson, S. E., Kahn, R. S., Khoury, J. & Lanphear, B. P. Racial Differences in Exposure to Environmental Tobacco Smoke among Children. *Environ. Health Perspect.* **113**, 362–367 (2005). 15743729 10.1289/ehp.7379 PMC1253766

[47] Little, R. J. et al. The Prevention and Treatment of Missing Data in Clinical Trials. *N. Engl. J. Med.* **367**, 1355–1360 (2012). 23034025 10.1056/NEJMsr1203730 PMC3771340

